# Effect of a 12-Week Multi-Exercise Community Program on Muscle Strength and Lipid Profile in Elderly Women

**DOI:** 10.3390/nu16060813

**Published:** 2024-03-13

**Authors:** Hee-Sook Lim, Tae-Hee Kim, Hyun-Joo Kang, Hae-Hyeog Lee

**Affiliations:** 1 Department of Gerontology, AgeTech-Service Convergence Major, Graduate School of East-West Medical Science, Kyung Hee University, Yongin 17104, Republic of Korea; limhsgeron@khu.ac.kr; 2 Department of Obstetrics and Gynecology, Soonchunhyang University College of Medicine, Bucheon 14584, Republic of Korea; 3 Department of Sports Medicine, Soonchunhyang University, Asan 31538, Republic of Korea

**Keywords:** exercise, aged, women, physical functional performance, muscle strength, sarcopenia

## Abstract

This study targeted elderly women over 60 years old (109 persons), divided them into an exercise group and a control group, and implemented a 12-week physical activity program for the exercise group. Body composition, muscle, blood tests, depression, quality of life (QoL), nutritional status, and physical strength were compared and analyzed. The physical activity program was organized through a consultative body of experts, was performed for about 60 min each time in the type and order of exercise appropriate for elderly women, and consisted of a combination of exercise using a band, gymnastics, and stretching. Changes in the muscle index and muscle mass before and after the program were selected as the primary efficacy evaluations. In the exercise group, waist circumference significantly decreased, and the muscle index significantly increased compared to the control group. The number of subjects who showed sarcopenia with a muscle index of 5.4 or less in the exercise group significantly decreased from 22 (38.6%) before program implementation to 13 (22.8%). According to the results of secondary effectiveness evaluation, high-density lipoprotein cholesterol and apolipoprotein (Apo) A were significantly increased in the exercise group compared to the control group, and Apo B, triglyceride, and c-reactive protein showed a significant decrease. Regular physical activity is very important for improving the health and QoL of elderly women, and as a result of applying a customized program, effects such as increased muscle index, improvement of sarcopenia, and improvement of blood lipid status were confirmed. Therefore, it is believed that the physical activity program developed through this study can be applied as a community program for elderly women.

## 1. Introduction

Korea’s population decline and aging are progressing very rapidly, and the working-age population is expected to continue to decline by nearly 50% over the next 30 years [1]. There are approximately 9.73 million people over the age of 65, and the population in their 70s and older is showing a reversal, exceeding the population in their 20s [2]. The elderly population ratio in Korea is also expected to reach 19.2%, entering a super-aging society in 2025, and 40% in 2050 [1,2]. The aging population structure has emerged as a more serious problem than the decline in population size itself. The average life expectancy for Korean men announced in 2024 is 86.3 years and, for women, 90.4 years, already exceeding the Organisation for Economic Co-operation and Development (OECD) average and the longest life expectancy for women in the world [2]. The increase in women’s average life expectancy is attributed to the development of medical technology, increased interest in health, and the spread of healthy lifestyle habits. However, although women’s life expectancy is longer than men’s, the prevalence of osteoporosis, depression, metabolic syndrome, and heart disease has recently steadily increased, making women more vulnerable to health issues [3,4]. Accordingly, the United States, Canada, and Australia are also implementing various health promotion policies for each women’s life cycle from a gender perspective at the government level, and the World Health Organization is also interested in women’s health and has selected key health indicators for each women’s life cycle [5]. In Korea, increased interest in women’s health has also led to changes in social awareness of the elderly, leading to active discussions on improving quality of life (QoL) in old age and successful aging [6]. Health issues are considered to be the area that has the most important impact on QoL in old age [6].

According to the results of a survey on women’s health awareness by life cycle recently announced by the Korea Centers for Disease Control and Prevention, arthritis was ranked first as a health problem most likely to occur in old age, followed by stroke, fractures, heart disease, and diabetes [7]. In addition, according to data from the National Health Insurance Corporation, Korea’s current medical expenses compared to GDP are approximately KRW 209 trillion, or 9.7%, which is higher than the OECD country average of 9.3% [8]. As chronic diseases become the main cause of death, aging and hospitalization in long-term care hospitals are increasing. This increase is greatly contributing to the increase in medical costs [9]. Therefore, to reduce elderly medical expenses, it is emphasized that chronic diseases should be prevented and converted to a community-centered health service [10]. Since Korea implemented the Long-Term Care Protection Act in July 2008, several programs have been developed in line with health promotion policies to manage elderly health issues in the community. Research was conducted in various fields, such as medicine, nursing, public health, and nutrition, and an integrated and multidisciplinary approach, rather than a simple medical approach, was attempted [11,12]. However, even though elderly women, who account for more than half of all elderly people, are a major target of elderly health problems due to their proportion alone and are vulnerable due to physical, psychological, and environmental factors, the healthcare service support system is still inadequate compared to that available to male elderly people [13]. In order to develop a policy on the physical health of older women, a multifaceted and comprehensive approach must be taken, considering psychological, sociological, and economic aspects. As part of this, a “Development and effectiveness evaluation of a community intervention research program for health promotion in elderly women” was carried out [14]. Currently in Korea, at a national management level, ‘Healthy 100-Year Exercise Class’ and ‘National Fitness 100′ programs are in place; although a ‘health promotion class’ is being operated, the actual participation rate is low, and the chronic disease management education program centered on the elderly group, which has been implemented at Seoul public health centers and community senior schools since 2007, is limited in effectiveness, as it is conducted in the format of large group lectures [15]. Therefore, community linkage is desirable for health care for the elderly, and the need for integrated management such as physical and emotional intervention is raised.

As such, it is very necessary to analyze the current status of community health promotion programs in preparation for the upcoming super-aging society and the increase in the proportion of elderly women. In addition, it is believed that research should be conducted to develop a health program that takes into account the social environment and physical health level of elderly women, prove its effectiveness, disseminate it as a customized program at community health centers, and expand the base. Through this study, the health level of elderly women was evaluated, a community complex program was developed, and a study was conducted to evaluate its usefulness. The precise aim of this study was to evaluate a program developed to be commonly deployable regardless of variations in the environment or facilities of local communities, with the goal of aiding in the improvement of elderly health.

## 2. Materials and Methods

### 2.1. Study Participants

This study evaluated changes in health promotion status after applying a physical activity program to elderly women living in Gyeonggi-do, especially changes in physical health, including sarcopenia, and mental health effects. Subjects included elderly women aged 60–75 who did not meet the exclusion criteria of disease (such as hypertension, diabetes, and hyperlipidemia) and who consented to participate in the study. Individuals with moderate-to-severe diseases that could pose a risk during moderate exercise (such as respiratory failure, arthritis, history of bone-related surgery, inability to walk, and severe conditions like stroke or cancer), those who engage in regular exercise at least three times per week for health management, those who had consumed muscle-enhancing supplements or health functional foods within one month prior to participating in the study, those taking medications (such as steroids or bone-joint drugs or diabetes or dyslipidemia medications), individuals aged 76 or older, and those consuming fewer than two irregular meals a day were all excluded. A total of 127 people were recruited, and excluding those who dropped out and missed the test, only the results of the final 109 people (57 in the exercise group and 52 in the control group) were analyzed. The study was approved by the Committee on Ethics of Soonchunhyang University (approval No. 1040875-201606-BR-002) according to the Declaration of Helsinki.

### 2.2. Multi-Exercise Program

At the exercise specialist consultative group, a resistance exercise program for elderly women was proposed after discussing the type and effectiveness of exercise. All subjects undergoing resistance band exercise were trained and supervised by a certified exercise specialist. Resistance bands and band loops (TheraBand; The Hygenic Corporation, Akron, OH, USA) were used to improve muscular fitness. Each exercise session consisted of a warm-up of 10 min, followed by a resistance training session (30–40 min), and was completed by a cool-down of 10 min; sessions occurred for 12 weeks. Before the start of the exercise, demonstrations were conducted and instructions were provided on designing a tailored exercise program. Following an adaptation phase of 4 weeks (1 set of 10–12 reps) using low resistance (yellow Thera-Band), exercise intensity was progressively augmented by increasing the resistance of the resistance band from yellow to red for weeks 5–8. From weeks 9 to 12, new exercises were learned and mixed with previously performed exercises. Exercise intensity (as indicated by band color) was set at a level that the patients perceived as “somewhat hard–hard”, which is equivalent to a 13–15 grade rating on the RPE scale according to the American College of Sports Medicine [16]. Additionally, the exercise volume was enhanced by increasing the number of sets from one to two. The progression rate was also based on individual improvements. For interest and exercise continuity, music preferred by the elderly was used. Each session was conducted in a safe and comfortable atmosphere. The movements and procedure of the resistance exercise program are as follows (Table S1).

### 2.3. Nutritional Assessment and Education

In order to effectively conduct this study and increase subject compliance, the same nutritional management methods for the elderly, nutritional status evaluation methods, and dietary therapy for chronic diseases were applied in both groups. The Mini Nutritional Assessment (MNA) was used to evaluate the subjects’ nutritional status [17]. The MNA consists of a total of 18 items, including meal intake, frequency of intake of each food, body mass index, and body circumference. Nutritional education and counseling were conducted once a month for a total of four sessions. The sessions included assessments of the participants’ nutritional status, focusing on key management priorities, appropriate portion sizes for the elderly, recommendations for regular and balanced nutrient intake, selecting healthy snacks and dining out, moderation in alcohol consumption, appropriate use of dietary supplements or alternative therapies, and methods for nutritional supplementation in case of malnutrition.

### 2.4. Variable

A survey was conducted on the study subjects’ age, age at menopause, education level, economic status, drinking status, usual stress level, and subjective health recognition. Demographic information variables were collected using standardized and structured survey instruments from the Korea National Health and Nutrition Examination Survey [18]. For anthropometric measurements, height, weight (InBody 720; Biospace, Seoul, Republic of Korea), and waist circumference were measured, and a whole-body scan was performed using dual-energy X-ray absorptiometry (DXA; Hologic Inc., Bedford, MA, USA) to accurately check body composition and muscle analysis. An X-ray bone density meter (Horizon W, Marlborough, New Zealand) was used. Using the results obtained through DXA, the skeletal muscle index (SMI) was calculated, and the presence or absence of sarcopenia was determined. 

For blood tests, blood was collected after fasting for 8 h before the test, and lipid tests (total cholesterol, low-density lipoprotein cholesterol [LDL-C], high-density lipoprotein cholesterol [HDL-C], apolipoprotein [Apo] A1, Apo B, and triglyceride [TG]) and inflammation-related tests (C-reactive protein [CRP]) were performed. 

The subjects’ QoL was assessed using the EuroQol 5-dimension 5-level, and a total of 5 response items were asked to determine whether there was any disruption or discomfort in 5 following areas: mobility, self-management, daily activities, pain/discomfort, and anxiety/depression. Depressive status was assessed using the Korean Geriatric Depression Scale 5-item (GDS-5) and the Geriatric Depression Scale Short Form Korea Version (GDSSF-K-15) [19]. The Korean Geriatric Depression Assessment Scale consists of five survey questions, and if the answer to even one of them was ‘yes’, an additional survey was conducted using the shortened geriatric depression scale. The shortened tool consists of 15 questions, and the score is calculated by answering ‘yes (1 point)’ or ‘no (0 points)’ to each question asking whether the subject has been depressed over the past week. The total score is 0–15 points. The higher the value, the more severe the depression. Out of 15 points, the depression evaluation classification score is 8, with 0 to 7 points being normal and 8 or more points being considered depressed. 

Physical activity was measured using the shortened version of the International Physical Activity Questionnaire (IPAQ), which includes information on vigorous physical activity, moderate physical activity, and walking physical activity over the past week, as well as time spent sitting. In this study, the *Fullerton* Functional *Fitness Test (FFT)*, a fitness assessment tool for the elderly, was used to confirm the effectiveness of the program. The FFT is a test developed to evaluate the independent living ability of elderly people and is known as the senior fitness test. The test is easy to perform, its stability for participants is guaranteed, the deviation is not severe, and reliability and construct validity have been proven. There are a total of 6 types of FFT evaluation items (standing up and sitting down on a chair, lifting dumbbells, walking in place for 2 min, sitting on a chair and bending forward, holding hands behind the back, and walking 244 cm), and each item measures the lower-body muscle strength and upper-body strength of the elderly. It is an item that evaluates physical strength, whole-body endurance, lower-body flexibility, upper-body flexibility, agility, and dynamic balance. Through before-and-after comparison, the degree of increase in muscle mass, strength, and vitality of elderly women can be evaluated.

### 2.5. Statistical Analyses

Data analyses were performed using the Statistical Package for SPSS version 25.0 (SPSS Inc., Chicago, IL, USA). Between-group differences in participants’ characteristics and secondary outcome measures were assessed using the Mann–Whitney U test for continuous variables and χ^2^ tests for categorical variables. *p*-values of less than 0.05 were used to denote statistical significance.

## 3. Results

### 3.1. Characteristics of Study Participants

The comparative results of general information and socioeconomic factors of the exercise group and control group are shown in Table 1. The average age of all subjects was 65.4 years old; the average of the exercise group was 66.8 years old, and that of the control group was 63.8 years old. The average age of the exercise group was slightly significantly higher. Regarding the level of education, the average of all subjects was high school graduate or less (96.3%), and in terms of overall economic level, 1.8% were classified as upper, 22.9% as upper-middle, 53.2% as lower-middle, and 22.0% lower class, with no difference between the two groups. The proportion of those who drank alcohol was 22.9% overall (17.5% in the exercise group and 28.8% in the control group) and higher in the control group. In terms of perceived stress, 0% reported feeling a lot, 58.7% reported feeling a little, and 41.3% reported not feeling it at all, with no significant difference between the two groups. When asking about subjective health status recognition, the proportions of those who responded, ‘not healthy’ and ‘very unhealthy’ were 10.5% and 0.0% in the exercise group and 19.2% and 1.9% in the control group, respectively, so the proportions in the control group were slightly higher. There were no significant differences between the two groups in baseline prevalence of chronic diseases, which is an important indicator for comparing health outcomes.

### 3.2. Comparison of Body Composition and Muscle Strength

The results of changes in body composition and muscle condition, which can be considered as the primary efficacy evaluation indicators, are shown in Table 2. As time passed, between groups, the body fat percentage of the exercise group significantly decreased, and the total muscle mass increased. In the case of appendicular skeletal muscle mass, there was no significant difference between groups, but significant changes were observed in SMI and sarcopenia participants across both time and groups.

### 3.3. Comparison of the Blood Test

The results of comparing blood tests are shown in Table 3. In the exercise group, total cholesterol, LDL-C, CRP, and blood pressure decreased. In particular, TG decreased significantly by an average of 54.3 mg/dL, and HDL-C and Apo A increased. In contrast, in the control group, LDL-C and Apo B increased. There was a significant change in CRP, but it was within the normal range in both groups.

### 3.4. Comparison of QoL, Depression, and Physical Activity

There was no significant change in depressive state and QoL, which can greatly affect daily life, following the implementation of the program. In the exercise group, walking exercise increased, and all basic physical fitness items, including muscular fitness of the lower body, muscular fitness of the upper body, general endurance, flexibility of the lower body, flexibility of the upper body, and agility and balance, were statistically significantly increased (Table 4). 

## 4. Discussion

This study was conducted to develop a physical activity program for elderly women as part of social support to lead a healthy life, verify its validity, and use it as a community program. A final total of 109 women who were relatively healthy or had mild chronic disease and did not exercise regularly were separated into an exercise group and a control group, and the before-and-after results of the program were compared and analyzed. In the results before program implementation, there were no significant differences in anthropometry, muscle condition, biochemical tests, depressive state, or QoL between the two groups. In other words, the program could be applied, and the allocation of subjects was balanced. After implementing a 12-week physical activity program, the anthropometric measurements and muscle status of the two groups were compared. In the exercise group, waist circumference, body fat percentage, and body fat mass decreased, while limb muscle mass, muscle index, and total muscle mass significantly increased, and in the control group, muscle index decreased. Looking at previous studies [18] that conducted physical activity targeting elderly women, it was found that gymnastics programs had a positive effect on cardiorespiratory function and health stamina. Pilates exercise improved body weight and muscle strength, and Silverobics improved active stamina [20]. The loss of muscle mass during the normal aging process is called sarcopenia, and when it is accompanied by obesity, it is called sarcopenic obesity (SO) [21]. It is explained that this is more dangerous in the elderly because it causes health problems at the same time. In a previous study that conducted a 16-week complex exercise program in elderly women aged 65 years or older, dividing them into SO and non-SO groups, body fat percentage in both groups significantly decreased, and body muscle mass significantly decreased in the non-SO group [22]. In both groups, the body fat percentage of the subjects in this study was higher than the normal range, and the total muscle mass was low, so more than 50% of the subjects could be considered to have SO [22,23]. After implementing the program, it was observed that the body composition status of the exercise group changed positively. Studies including systematic literature reviews have also shown that elderly exercise intervention programs have a positive effect on various health conditions, such as muscle strength improvement and aging prevention [22,23,24,25]. 

In the case of blood tests, the exercise group showed an increase in HDL-C and Apo A levels and a significant decrease in TG and CRP compared to before the program. Regular aerobic exercise is known to help prevent and treat cardiovascular diseases by activating lipoprotein enzymes to break down neutral fat in the blood and improve HDL-C [26,27]. In particular, active elderly people over 65 years of age showed much better serum lipid status than inactive subjects. Apo interacts with phospholipids to help dissolve cholesterol esters and neutral fats and regulates the reactions of lipids and various enzymes [28,29]. The main lipoprotein of HDL-C is Apo A1, the main lipoprotein of LDL-C is Apo B, and the main lipoprotein of VLDL cholesterol is Apo C [28]. During exercise, Apo A-1 and Apo C-II increase, and Apo B and Apo C-III are known to either increase or decrease [28]. Nassef et al. [30] emphasized the importance of exercise by showing that the arteriosclerosis index, neutral lipids, and Apo B decreased, while HDL-C increased in the results of exercise for middle-aged women. CRP is an acutely reactive substance that increases due to inflammatory reactions and non-specific reactions of cells and tissue metabolism [31]. It is used as a standard for measuring disease activity and is closely related to cardiovascular disease. It has been reported that physical activity reduces the level of body inflammation and insulin resistance in the elderly, and a domestic study showed that CRP significantly decreased as a result of aerobic exercise and muscle resistance training in elderly people [32]. There are not many studies that have conducted in-depth analysis of Apo or CRP through exercise in elderly women, so comparison is difficult, but it is believed that this study obtained very meaningful results regarding the reduction in blood lipid levels [32,33]. After implementing the program, there was no significant change in depression in either group, and in terms of QoL, the QoL score in the exercise group showed a slight improvement, but there was no difference between the two groups. Additional in-depth analysis should be conducted on the factors that affect depression and cognitive decline, and considering the fact that there are limitations in proving the effectiveness of a short-term physical activity program alone, it is necessary to improve the comprehensive QoL of elderly women in the future. To achieve this, it is believed that a program that explores not only physical activity but also psychological and mental factors should be prepared. As a result of the elderly nutrition assessment, the risk of malnutrition was found to be high in both groups. Since it was applied and analyzed as a matter of regular and balanced meal intake, there may be differences from the malnutrition rate at a disease level, but when examining detailed factors, drug use, inadequate intake of protein foods, insufficient daily water intake, and nutritional status were observed. It can be seen that areas such as lack of awareness have a significant impact on malnutrition. After implementing the physical activity program, all six basic physical fitness items of the exercise group showed significant improvement. It is said that exercise has a positive effect on body composition and blood test status in the short term, as well as an effect on improving physical strength, such as cardiorespiratory endurance and muscle strength, in the long term. Cadore et al. [34] implemented a multi-exercise program among elderly people with an average age of 91 and showed improvement in strength and muscle mass. Binder et al. [35] conducted exercise training for 9 months with elderly people aged 78 or older with physical aging symptoms and reported results of exercise training for 9 months. It was explained that proper exercise should not be limited by age to keep pace with the aging population by gaining the effect of improving muscle mass and physical strength. There are many tools to measure physical strength, but this study used indicators recommended for elderly women, and other tools suitable for Koreans should be analyzed and applied in the future. In this study, a physical activity program was designed through a combination of various movements, but realistically, it was difficult to control and complete the exercise group over a short period of 12 weeks. In addition, because each elderly woman had some differences in individual exercise ability and health status, even if it was planned by an expert, the most difficult part was managing to perform the various movements the same every time and not forget the movements even when not exercising. Furthermore, the results of the 12-week short-term study do not demonstrate long-term sustainability. In other words, more definitive and long-term research is needed to determine whether quality of life and psychosocial factors are reflected over an extended period. Additionally, to evolve into a multi-faceted program suitable for the elderly, future research should be continued with structured and long-term models, as well as differentiated programs. However, we put a lot of effort into increasing muscle mass through exercise by moving as many different parts of the body as possible and were able to complete the program safely by deploying safety personnel and exercise assistants on site. Physical strength is recognized as a very important factor not only for the elderly but also for all age groups, and the country is focusing on improving the physical strength of the people by developing various contents, such as the physical fitness certification project to welcome the era of national health to 100 years. It is believed that improving basic physical strength through exercise can help extend the healthy lifespan of the elderly.

## 5. Conclusions

Regular physical activity is very important for improving the health and QoL of elderly women, and as a result of applying a customized program, effects such as increased muscle index, improvement of sarcopenia, and improvement of blood lipid status were confirmed. This was a very meaningful study that yielded results on the various benefits of a short-term physical activity program, and it is believed that more attention should be devoted to the complex management of elderly women’s health.

## Figures and Tables

**Table 1:** Social characteristics and health-related factors of the subjects.

	Total (*n* = 109)	Control Group (*n* = 52)	Exercise Group (*n* = 57)	*p* Value
Age (years)	65.4 ± 4.3	63.8 ± 3.8	66.8 ± 4.3	**<0.001**
Menopausal age (years)	50.8 ± 4.6	51.0 ± 5.0	50.7 ± 4.3	0.298
Educational level				
≤Middle school	68 (62.4)	64 (65.4)	34 (59.6)	0.619
High school	37 (33.9)	16 (30.8)	21 (36.8)	
≥College	4 (3.7)	2 (3.8)	2 (3.6)	
Economic status				
High	2 (1.8)	0 (0.0)	2 (3.5)	0.309
Middle–high	25 (22.9)	13 (25.0)	12 (21.1)	
Middle–low	58 (53.2)	25 (48.1)	33 (57.9)	
Low	24 (22.0)	14 (26.9)	10 (17.5)	
Drinking				
Drinker	25 (22.9)	15 (28.8)	10 (17.5)	0.374
Past drinker	7 (6.5)	5 (5.8)	4 (7.0)	
Non-drinker	77 (70.6)	34 (65.4)	43 (75.4)	
Usual stress level				
A lot	0 (0.0)	0 (0.0)	0 (0.0)	0.710
A little	64 (58.7)	31 (59.6)	33 (57.9)	
None	45 (41.3)	21 (40.4)	24 (42.1)	
Subjective health recognition				
Very healthy	4 (3.7)	2 (3.8)	2 (3.5)	0.522
Healthy	88 (80.7)	39 (75.0)	49 (86.0)	
Unhealthy	16 (14.7)	10 (19.2)	6 (10.5)	
Very unhealthy	1 (0.9)	1 (1.9)	0 (0.0)	
Diseases				
Diabetes	10 (9.2)	6 (11.5)	4 (7.0)	0.628
Hypertension	19 (17.4)	12 (23.1)	7 (12.3)	0.218
Hyperlipidemia	12 (11.0)	9 (17.3)	3 (5.3)	0.089

The data are presented as mean ± standard deviation or N (%). The *p* values were obtained using an χ^2^ test for categorical variables and an independent *t*-test for continuous variables. Bold values indicate statistical significance.

**Table 2:** Comparison of body composition and muscle strength variables at baseline and 12 weeks post intervention in the control and exercise groups.

	Control Group (*n* = 52)		Exercise Group (*n* = 57)		Effects (*p* Value)		
	Baseline	12 Weeks	Baseline	12 Weeks	Time	Group	Time × Group
Body mass index (kg/m^2^)	25.4 ± 3.6	25.6 ± 3.5	24.6 ± 3.0	24.6 ± 2.9	0.643	0.651	0.608
Waist circumference (cm)	33.5 ± 3.3	34.1 ± 3.6	33.1 ± 3.3	32.1 ± 4.6	0.226	0.150	0.114
Body fat (%)	42.2 ± 4.0	41.9 ± 4.0	41.0 ± 3.7	39.4 ± 3.6	0.368	**0.011**	**0.035**
Lean body mass (kg)	32.7 ± 3.6	32.3 ± 3.1	32.3 ± 3.7	32.2 ± 3.7	0.165	0.092	0.132
Total muscle mass (kg)	14.2 ± 1.8	14.1 ± 2.1	13.9 ± 2.1	14.3 ± 1.8	**0.008**	**0.015**	**0.036**
Appendicular skeletal mass (kg)	12.9 ± 1.6	12.8 ± 1.7	12.6 ± 1.9	13.0 ± 1.6	0.270	**0.013**	0.104
Skeletal muscle index (kg/m^2^)	5.4 ± 0.6	5.3 ± 0.7	5.3 ± 0.7	5.6 ± 0.7	**0.007**	**0.001**	**0.010**
Sarcopenia	30 (57.7)	27 (51.9)	33 (57.9)	22 (38.6)	**0.027**	**0.016**	**0.004**
Risk of malnutrition based on MNA							
Normal	24 (46.2)	14 (26.9)	21 (36.8)	11 (19.3)	0.053	0.267	0.118
At malnutrition risk	28 (53.8)	38 (73.1)	36 (63.2)	46 (80.7)			
Malnutrition	0 (0.0)	0 (0.0)	0 (0.0)	0 (0.0)			

The data are presented as mean ± standard deviation or N (%). Better results were observed in the control group than the exercise group before and after the exercising period (*p* < 0.05). Bold values indicate statistical significance. MNA, Mini Nutritional Assessment.

**Table 3:** Comparison of lipid profile and cytokine variables at baseline and 12 weeks post intervention in the control and exercise groups.

	Control Group (*n* = 52)		Exercise Group (*n* = 57)		Effects (*p* Value)		
	Baseline	12 Weeks	Baseline	12 Weeks	Time	Group	Time × Group
Total cholesterol (mg/dL)	99.4 ± 37.4	98.6 ± 34.9	87.8 ± 30.9	82.8 ± 36.6	0.824	0.253	0.214
LDL cholesterol (mg/dL)	115.9 ± 34.1	117.0 ± 32.2	103.9 ± 102.7	102.7 ± 31.1	0.761	0.646	0.758
HDL cholesterol (mg/dL)	50.2 ± 11.1	50.9 ± 11.2	52.5 ± 12.8	58.9 ± 12.5	**0.044**	**0.002**	**0.008**
Triglyceride (mg/dL)	154.0 ± 76.7	145.6 ± 80.6	171.9 ± 117.2	117.6 ± 45.9	**<0.001**	**<0.001**	**<0.001**
Apolipoprotein A (mg/dL)	143.0 ± 19.2	141.1 ± 20.5	148.2 ± 20.6	155.7 ± 21.0	**0.007**	0.108	**0.039**
Apolipoprotein B (mg/dL)	95.2 ± 21.4	104.2 ± 25.9	86.0 ± 18.1	84.7 ± 19.5	0.282	0.147	0.320
C-reactive protein (mg/dL)	0.10 ± 0.31	0.22 ± 0.32	0.23 ± 0.21	0.10 ± 0.11	**0.011**	**0.005**	**0.030**

The data are presented as mean ± standard deviation or N (%). Better results were observed in the control group than the exercise group before and after the exercising period (*p* < 0.05). Bold values indicate statistical significance. LDL, low-density lipoprotein; HDL, high-density lipoprotein.

**Table 4:** Comparison of quality of life, depression, and physical activity at baseline and 12 weeks post intervention in the control and exercise groups.

	Control Group (*n* = 52)			Exercise Group (*n* = 57)		
	Baseline	12 Weeks	*p* Value	Baseline	12 Weeks	*p* Value
EQ-5D score	0.89 ± 0.12	0.89 ± 0.08	0.797	0.89 ± 0.10	0.90 ± 0.08	0.206
GDS	11 (21.2)	15 (28.8)	0.386	11 (19.3)	9 (15.8)	0.773
GDSSF-K						
Normal	45 (86.5)	43 (82.7)	0.789	51 (89.5)	51 (89.5)	1.000
Depressed	7 (13.5)	9 (17.3)		6 (10.5)	6 (10.5)	
Physical activity						
Vigorous activity (day/week)	3.1 ± 1.8	2.5 ± 1.0	0.584	3.5 ± 1.7	3.5 ± 1.2	0.479
Moderate activity (day/week)	3.5 ± 1.6	3.0 ± 1.6	0.581	4.2 ± 1.6	3.8 ± 1.5	0.608
Walking (day/week)	4.4 ± 1.9	5.2 ± 2.0	0.072	5.1 ± 1.7	6.2 ± 1.0	**<0.001**
Physical fitness						
Muscular fitness of lower body (times/30 s)	18.0 ± 2.8	19.6 ± 3.4	0.716	17.7 ± 3.4	25.1 ± 5.9	**<0.001**
Muscular fitness of upper body (times/30 s)	19.1 ± 3.7	19.9 ± 5.6	0.883	20.8 ± 4.3	31.9 ± 26.8	**<0.001**
General endurance (times/2 min)	121.4 ± 11.2	128.6 ± 15.3	0.465	119.4 ± 17.7	137.0 ± 25.7	**<0.001**
Flexibility of lower body (cm)	10.2 ± 5.4	14.3 ± 2.8	0.041	11.4 ± 9.0	21.0 ± 6.7	**<0.001**
Flexibility of upper body (cm)	1.6 ± 2.1	1.3 ± 4.6	0.154	−3.2 ± 9.1	0.2 ± 8.0	**<0.001**
Agility and balance (s)	7.0 ± 2.3	6.8 ± 1.9	0.220	6.4 ± 1.2	5.6 ± 1.0	**<0.001**

The data are presented as mean ± standard deviation or N (%). Better results were observed in the control group than the exercise group before and after the exercising period (*p* < 0.05). Bold values indicate statistical significance. EQ-5D, EuroQol-5 dimension; GDS, Geriatric Depression Scale; GDSSF-K, Geriatric Depression Scale Short Form-Korean Version.

## Data Availability

The data are available with approval from the Korea Disease Control and Prevention Agency.

## Supplementary Materials

The following supporting information can be downloaded at: https://www.mdpi.com/article/10.3390/nu16060813/s1, Table S1: Movements and procedure of the resistance exercise program.
