# Supplementary material for: Effect of a 12-Week Multi-Exercise Community Program on Muscle Strength and Lipid Profile in Elderly Women
Source: Nutrients. 2024 Mar 13;16(6):813. doi: 10.3390/nu16060813 (PMC10976123; doi:10.3390/nu16060813)
Supplement: Supplementary file 1 [file nutrients-16-00813-s001.zip › nutrients-2879517-supplementary.pdf]

Table S1: Movements and procedure of the resistance exercise program.

No.	Week	Exercise	Main Muscle
1	1wk	Hip contraction	Gluteus maximus, Gluteus medius, Piriformis, Iliopsoas
2		Hip external rotation	Gluteus maximus, Piriformis, Gemellus, Obturator
3		Rhomboideus exercise	Rhomboideus
4	2wk	Hip contraction	Gluteus maximus, Gluteus medius, Gluteus minimus
5		Hip abduction	Iliopsoas, Psoas major, Sartorius, Gluteus maximus, Piriformis, Gemellus, Obturator
6		Hip abduction	Iliopsoas, Psoas major, Gluteus maximus
7	3wk	Hip contraction	Gluteus maximus, Erector spinae muscle
8		Back muscle exercise Lower body exercise	Erector spinae muscle, Biceps femoris, Semimembranosus, Adductors, Gastrocnemius
9		Back muscle exercise	Erector spinae muscle, Biceps femoris
10	4wk	Abdominal exercise	Rectus abdominis, Transversus abdominis, Rectus femoris
11		Abdominal exercise	Rectus abdominis, External oblique, Internal Oblique, Transversus abdominis
12		Hip contraction	Gluteus maximus, Biceps femoris, Semitendinous, Semimembranosus
13	9wk	Shoulder exercise	Deltoid
14		Shoulder exercise	Deltoid
15	10wk	Upper body exercise	Biceps brachi
16		Upper body exercise	Triceps brachi
17	11wk	Dead lift	Biceps femoris
18		Squat	Quadriceps femoris
19	12wk	Band One Arm Row	Latissimus dorsi
20		Band Seated Row	Latissimus dorsi
21		Band Leg press	Quadriceps femoristka

No. 1-12, 3 movements per week for 1 to 4 weeks and repeat for 5 to 8 weeks; No. 13-21, 2 to 3 movements per week for 9 to 12 weeks and repeat. wk; week.
